# Identification and Analysis of Labor Productivity Components Based on ACHIEVE Model (Case Study: Staff of Kermanshah University of Medical Sciences)

**DOI:** 10.5539/gjhs.v7n1p315

**Published:** 2014-12-15

**Authors:** Arash Ziapour, Alireza Khatony, Neda Kianipour, Faranak Jafary

**Affiliations:** 1 Vice chancellery for Research and Technology, Kermanshah University of Medical Sciences, Kermanshah, Iran; 2 Social Development and Health Promotion Research Center, Kermanshah University of Medical Sciences, Kermanshah, Iran; 3 Department of Environmental Health Engineering, Kermanshah University of medical sciences, Kermanshah, Iran; 4 Kermanshah School of Nursing and Midwifery, Kermanshah University of Medical Sciences, Kermanshah, Iran

**Keywords:** productivity, labor, Kermanshah University of Medical Sciences, staff, ACHIEVE model

## Abstract

Identification and analysis of the components of labor productivity based on ACHIEVE model was performed among employees in different parts of Kermanshah University of Medical Sciences in 2014. This was a descriptive correlational study in which the population consisted of 270 working personnel in different administrative groups (contractual, fixed- term and regular) at Kermanshah University of Medical Sciences (872 people) that were selected among 872 people through stratified random sampling method based on Krejcie and Morgan sampling table. The survey tool included labor productivity questionnaire of ACHIEVE. Questionnaires were confirmed in terms of content and face validity, and their reliability was calculated using Cronbach’s alpha coefficient. The data were analyzed by SPSS-18 software using descriptive and inferential statistics. The mean scores for labor productivity dimensions of the employees, including environment (environmental fit), evaluation (training and performance feedback), validity (valid and legal exercise of personnel), incentive (motivation or desire), help (organizational support), clarity (role perception or understanding), ability (knowledge and skills) variables and total labor productivity were 4.10±0.630, 3.99±0.568, 3.97±0.607, 3.76±0.701, 3.63±0.746, 3.59±0.777, 3.49±0.882 and 26.54±4.347, respectively. Also, the results indicated that the seven factors of environment, performance assessment, validity, motivation, organizational support, clarity, and ability were effective in increasing labor productivity. The analysis of the current status of university staff in the employees’ viewpoint suggested that the two factors of environment and evaluation, which had the greatest impact on labor productivity in the viewpoint of the staff, were in a favorable condition and needed to be further taken into consideration by authorities.

## 1. Introduction

Human resources are one of the most important components and assets of any organization (Hanafi, 2010). Growth and development of human resources, and promoting the skills, creativity and knowledge of the workforce at all levels of the organization has been considered a strategic priority for managers from 1990s (Bordbar, 2002). Labor productivity is one of the essential factors for countries to achieve the scientific and industrial progress and ultimately economic development. Improving the quality of human resources is essential for development since the foundation will be human development, and any planning to develop human resources has a fundamental and decisive role (Martin, 1998). Therefore, economic development is not achieved without making the development of human resource a priority because only in a long-term process a bright future trajectory will be realized through planning and a comprehensive approach based on detailed information from the past (Sahai, 2005; Lyta, 1998). Productivity of human resources in health care organizations will have even greater importance because in addition to their routine duties, they face special missions and need to be ready to confront crises (Bahadory, 2010). Efficiency in the healthcare services sector is almost a new subject, since most measurements have been done in the private sector, industries and factories (Mahram, 2011). Also, evaluation of the efficiency of health care, especially its effectiveness factor is not as easy as that of a manufacturing company or business and it is very complex, this is because of the different nature of healthcare services than goods. In addition, services do not have the capacity of being stored (Bernard, 1996). The output cannot easily be converted to numbers in measuring productivity of the health sector (Faqihi, 1998). Today, the reduction of productivity and labor recession are a challenge for some health organizations because of lack of efficient use of human resources. So, identifying and strengthening the predisposing components of labor productivity can enhance the efficiency of the whole organization (Mahram, 2011). Therefore, the ACHIEVE model can help managers to determine difficulties in the performance. Hersey and Blanchard have considered seven factors affecting the performance of human resources that are planned in order to help managers to determine the causes of performance problems and to create the change strategies in order to solve these problems (Rezayian, 2002). These factors include:1-ability (knowledge and skills), 2-clarity (perceived or imagined role), 3-help (organizational support), 4-incentive (motivation or desire), 5-evaluation (training and performance feedback), 6-validity (valid and legal practice of employees) and 7-environment (environmental fit). According to official statistics published by the scientific community, the working hours in Japan, South Korea and America include 49 to 60, 54 to 72 and 36 to 40 hours per week, respectively; however, the working hours in Iran range from 6 to 9 hours per week (Bahadory, 2010; Taheri, 2006). Identification of the factors affecting the promotion of labor productivity is the main aim of all researchers in this field. All researchers believe that a single factor cannot account for the development in labor productivity, but a combination of different factors interfere with promotion of labor productivity. Reports show that the labor productivity index in Iran is very low compared to East Asia and countries in the region (Taheri, 2009).

Human resource productivity is influenced by a variety of factors, and several studies have been done in this area. Alvani (2001) developed a model for labor productivity in which the components of leadership, motivation, experience, creativity and innovation, education, competition and characteristics of the population were identified as the most important factors affecting productivity (Alvani, 2001). Mehrabian et al. (2011) showed that organizational culture, motivational factors, environmental conditions, empowerment and leadership style were the most important factors in improving labor productivity in Guilan University of Medical Sciences, indicating the organizational culture to have a greater impact than the other factors. In another study by Bahadori et al. (2013), it was conducted that the most important factors affecting productivity included ability, job clarity, job performance, evaluation, motivation and environmental factors (Bahadori et al., 2013). In another study conducted by Bs & Associates (2012), it was revealed that electronic health records, as one of the variables of organizational support, had an important role in increasing productivity. The current statistics show that, contrary to industrial and commercial organizations, universities and medical education institutions rarely review the factors and methods to promote productivity and human resource in Iran, and researches conducted by industrial organizations are not applicable to health and education centers (Jordan, 1994; Bahadori, 2010). The studies conducted in other countries cannot be generalized to Iranian organizations due to cultural, social and economic differences. Thus, as the highest ranking scientific centers in society, universities and higher education institutions require useful tool in determining productivity of human resources in order to perform their responsibilities and to improve and assess their efficiency (Khodayari, 2008). So, the researchers made an attempt to assess views of the staff of Kermanshah University of Medical Sciences about the human performance using ACHIEVE Model in order to provide a ground for development of human resources by policy makers, decision makers and managers.

## 2. Materials

This study was a descriptive correlational study in which the statistical society included all working personnel in different administrative groups (formal and informal contract) at Kermanshah University of Medical Sciences in 2014. The samples including 270 patients (132 men and 138 women) were selected using stratified random sampling based on the Krejcie and Morgan sampling table. Due to the heterogeneous nature of the samples, stratified random sampling method was applied through which168 contractual, 50 informal and 49 formal staff were selected. Data collection tools included a demographic questionnaire used to collect information about gender, age, marital status, education level, and work experience and a labor productivity questionnaire based on Achieve model was used in 26-item standard test based on Hersey and Goldsmith Model (Hersey & Goldsmith, 1980). It comprised of seven dimensions, including ability (knowledge and skills) (3 items), clarity (perceived or imagined role) (4 items), help (organizational support) (3 items), incentive (motivation or desire) (4 items), evaluation (education and performance feedback) (4 items), validity (valid and legal practice of the staff) (4 items), environment (environmental fit) (3 items), which was rated based on a five-point Likert scale; very low (1), low (2), somewhat (3), high (4) very high (5). The validity of the questionnaire was confirmed via face and content validity as well as expert opinion and the reliability was confirmed using Cronbach’s alpha coefficient (r2=0.93). The validity indices of this questionnaire reported in the studies carried out by Farshadfar (2009), Bordbar (2012), Allah Verdi et al. (2010), Bahadori (2013), Shariatmadari et al. (2012), Musa Zadeh et al. (2011), Mehrabian et al. (2011), Hatami et al. (2011), Sabkvar et al. (2010), Hedayati et al. (A. 2009), Nassiripuor et al. (2009), Vaezi et al. (2011) and Hedayati et al. (2011b) were 0.80, 0.87, 0.93, 0.70, 0.85, 0.91, 0.89, 0.89, 0.81, 0.81, 0.81, 0.97 and 0.86, respectively, and its Cronbach’s alpha coefficient was obtained greater than 0.7. This coefficient in the current study for labor productivity was 0.81. Since the closer this number is to one the reliability will in crease, this index indicated a high reliability owing to the internal consistency of the questions in the given questionnaire.

The questionnaires were distributed among the staff of the university and necessary explanations were given about the objectives of the research. The researchers tried to be present to answer questions as they arose and to provide the necessary instructions. Ethical considerations such as taking informed consent from the staff to participate in the research, impartiality and avoidance of certain bias and confidentiality and accuracy of information obtained from questionnaires were taken into account by the researchers. Data were analyzed by SPSS software (version 18, SPSS Inc., Chicago, IL) using descriptive statistics of distribution, percentage, standard deviation and inferential statistics of Pearson correlation coefficient.

## 3. Results

The descriptive findings showed that the mean labor productivity was moderate to high. Also, from the 270 university staff, 120 people (44.4%) were male and 150 people (55.6%) were female. In terms of age, 43 (15.9%) respondents were aged less than 30 years, 108 people (40%) were aged 31 to 40 years, 98 patients (36.3%) were in the age range of 41 to 50 years and 21 people (7.8%) were more than 50 years old. The highest age frequency was 31 to 40 years (108 patients), and the lowest age frequency was50 years (21 patients). In terms of education, 28 (10.4) people had diploma degree, 32 (11.9) staff had associate degree, 138 (51.1) respondents had bachelor degree and 72 (26.7) people have master degree and higher. The largest number was reported for bachelor degree. In terms of work experience, 41 people (15.2%) had 5 years of experience, 47 people (17.4%) between 5 to 10 years, 66 people (24.4%) between 10 to 15 years, 57 people (21.1%) between 15 to 20 years, and 59 people (21.9%) had ≥20 years of experience.

As shown in Table 1, the results showed the highest mean score among the seven labor productivity scores for the environment (environmental fit) (mean=4.10, SD=0.63007) and the lowest mean score for the ability (knowledge and skills) (mean=3.49, SD=0.88246). The findings also showed all indicators of labor productivity to be moderate to high. The overall mean and standard deviation of labor productivity of employees were 26.54 and 4.34774, respectively.

**Table 1:** Mean, standard deviation, minimum score, maximum score and respondents’ rank in the variables

Statistical indicators Variables		Mean	Standard deviation	Rank
**Variables**
	Environment (environmental fit)	4.10	0.63007	First
	Evaluation (training and performance feedback)	3.99	0.56826	Second
	Validity (valid and legal practice of employees)	3.97	0.60731	Third
	Incentive(motivation or desire)	3.76	0.70153	Fourth
	Help(organizational support)	3.63	0.74693	Fifth
	Clarity (perceived or imagined role)	3.59	0.77738	Sixth
	Ability (knowledge and skills)	3.49	88246.0	Seventh
	Labor productivity	26.54	4.34774	-

As shown in Table 1, the results showed the highest mean score among the seven labor productivity scores for the environment (environmental fit) (mean=4.10, SD=0.63007) and the lowest mean score for the ability (knowledge and skills) (mean=3.49, SD=0.88246). The findings also showed all indicators of labor productivity to be moderate to high. The overall mean and standard deviation of labor productivity of employees were 26.54 and 4.34774, respectively.

The results of correlation coefficient test presented in Table 2 shows a significant relationship between labor productivity and demographic variables of experience, age and education at the level of 1% (p <0.01). So, the research hypotheses were confirmed. According to the data in Table 2, there was no significant relationship between labor productivity and gender (p>0.01and R=0.029). Thus, the hypothesis was rejected. Moreover, among the demographic variables, work experience and sex with ratios of 0.192 and 0.029 had the strongest and the weakest relationship with labor productivity, respectively.

**Table 2:** Correlation coefficients of demographic variables and labor productivity

Hypothesis	Independent variable	Dependent variable	Frequency	Correlation coefficient	Sig (2-tailed)
1	Experience	Labor productivity	270	0.192**	0.001
2	Age	Labor productivity	270	0.181**	0.003
3	Education	Labor productivity	270	0.137**	0.024
4	Sex	Labor productivity	270	0.029**	0.632

** Correlation is significant at 0.01 (2-tailed).

## 4. Discussion

This study was conducted to identify and analyze labor productivity components based on ACHIEVE model among employees in different parts of Kermanshah University of Medical Sciences and Health Services in 2014.

The results of this study showed the highest mean score for environment (environmental fit) among the seven factors of labor productivity of employees, and the lowest mean score for ability (knowledge and skills), that show moderate to high levels for productivity of human resources. The environmental indicator was in a better position than the other indicators, which shows a low level of discrimination among employees. Trust and honesty among the staff and considering productivity in the workplace obtained a good level. These findings are in line with the findings of Nassiri pour et al. (2009) and Hedayati et al. (2009) about the mean efficiency of the staff at studied hospitals that showed a desirable level for the seven dimensions of productivity indices. The findings obtained from the classification of labor productivity indices showed that these dimensions (Table 1) affected the productivity of staff in Kermanshah University of Medical Sciences. Secondly, these components may have higher degrees of importance to substantially increase human resources productivity. Thus, according to mean scores obtained from data analysis. the factors affecting the productivity included environment (environmental fit), evaluation (education and performance feedback), validity (valid and legal practice of employees), incentive (motivation or desire), help (organizational support), clarity (perceived or imagined role) and ability (knowledge and skills).

Top of Form

The findings of the study carried out by AeinParast, entitled factors affecting the human resources efficiency at the Ministry of Health from the perspective of employees showed that some factors such as ability, knowledge, motivation, performance evaluation and in situational support were effective in enhancing labor productivity (AeinParast, 2000). Also, Mohammadi performed a research and came to this conclusion that the staff training, use of technology, implementation of motivational policy, and proper assessment of staff performance influenced productivity (Mohammadi, 2000), that is in line with the findings of this research. Moreover, the classification of factors influencing labor productivity based on the results of Hersey and Kenneth is as follows: ability (knowledge and skills), help (organizational support), evaluation (training and performance feedback), environment (environmental fit) and validity (valid and legal practice of employees). Some of these factors are similar to thecomponents reported in this research (Hersey & Kenneth, 1998). Furthermore, Wichian S, Wongwanich S, and Bowarn kiti wong investigated the factors influencing the research productivity of faculty members in state universities. They identified the personal characteristics of the researcher, in situational support for research and environmental conditions as the most important components to improve the research productivity of faculty members (Wichian, 2009). It can be argued that the results of the recent research are similar to the study conducted by Wichian in terms of the effect of environmental conditions on labor productivity. Also, according to a study conducted in 2008, the favorable working environment led to enhancement of productivity, which is compatible with the findings of the present study (Litvak, 2008). In another study conducted in 2011 on Iranian nurses, it was revealed that nurses with low productivity levels were not satisfied with their quality of working life, representing such components as working environment and organizational support that were high lighted in this study (Nayeri, 2008). In a research studying the factors affecting labor productivity among employees of Tarbiat Modares University, Jamalinejad (2001) found that environment was an important factor to improve labor productivity, which is consistent with the results of present study in terms of the effect of environmental conditions. Askari (2005) studied the factors influencing labor productivity in Fulad Mobarake Company and reported human resources training as a factor improving labor productivity. Although training can be introduced as a method for staff empowerment, it is not the only way of empowerment, which is in contrast with results of the present study.

In this study there was a significant relationship between the level of education and staff efficiency, the higher the education level of employees was the higher their tendency to increase productivity was, which is consistent with the results of Ostovar et al. (2003). Also, the results of ANOVA and correlation coefficient suggested that age, education and work experience significantly affected the labor productivity. Also the findings of the study by Bordbar evaluating the productivity of human resources in the industrial sector showed that age, education and work experience affected the efficiency of human resources (Bordbar, 2008).

## 5. Conclusion

The current study indicated that efficiency of human resource was desirable among the university staff. Given the critical role of employees’ performance in organizations, it is important to recognize the factors affecting the performance and productivity of the personnel.

Factors such as environment, evaluation, validity, incentive, support, clarity and ability had the maximum importance, respectively to enhance labor productivity in medical university staff. There fore, it is essential that administrators provide adesir able ground for their employees to improve productivity at different levels in the organization.

## Recommendations

Training courses are recommended to enhance labor productivity, to in crease innovation and to create a healthy environment among the university staff.
